# Valorizing Press Cakes as Ingredients in Textured Vegetable Proteins: Processing, Structure, and Texture

**DOI:** 10.1111/1750-3841.70471

**Published:** 2025-08-20

**Authors:** Lallinger Luise, Alejandra Maria Torres Gomez, Alejandra Maria, Niksch Jonas, Rauh Cornelia

**Affiliations:** ^1^ Department of Food Biotechnology and Food Process Engineering, Institute of Food Technology and Food Chemistry Technische Universität Berlin Berlin Germany

## Abstract

In this study, press cakes (PCs)—the by‐products from almond, coconut, flaxseed, pumpkin seed, rapeseed, and sunflower oil production—were used to create textured vegetable proteins (TVPs) that offer both nutritional and sustainability benefits. Several technological and textural properties were measured to evaluate the potential of the PCs for use as ingredients in meat alternatives.

After raw material characterization, 15 PC flours were mixed with pea protein isolate at three levels (25%, 45%, and 50–100% PC, depending on composition) and low‐moisture extruded in a pilot‐scale co‐rotating twin‐screw extruder (11% feed water content, 150°C barrel temperature, 10.18 kg/h throughput). Extrusion response parameters were recorded. The structure and texture of the resulting extrudates were analyzed by light microscopy, fracture test, and texture profile analysis. Finally, the PC‐TVPs were compared to reference products and meat.

Oil and protein content are the most important factors influencing processing and TVP properties because they reduce shear forces and cross‐linking. The decrease in protein network stability seen as PC content increases results in denser TVPs with smaller pores and thicker lamellae. These TVPs—especially those made with sunflower seed, flaxseed, and pumpkin seed PCs—are more chewy and less spongy than higher protein TVPs, making the texture more meat‐like.

Press cakes possess the techno‐functional properties to (partially) replace protein isolates or concentrates in TVP production, thereby improving the nutritional profile, texture, and sustainability of meat alternatives.

**Practical Applications:**

Technological insights gained from this study will facilitate the production of TVPs from press cakes in new plant‐based products. Drawing on the results of this study, producers will be able to choose press cake ingredients and concentrations to achieve the desired textural properties while diversifying their raw materials. Starting from the specified extrusion and formulation conditions indicated here, future research can further optimize processes and TVP properties.

## 1 Introduction

The replacement of protein‐rich products of animal origin, such as meat, with corresponding plant‐based alternatives has proven an effective means of reducing the environmental and climate footprints of global food production while also promoting consumer health (Alcorta et al. 2021; Clark et al. 2019; Poore and Nemecek 2018; Schösler et al. 2012). The use of varied and minimally processed protein sources in plant‐based alternatives would be optimal for a sustainable food supply chain (Singh et al. 2022). However, concentrates and isolates from wheat and pulses (soy, pea) are the most common protein ingredients used in meat alternative products due to their well‐defined processing properties, weak flavor, light color, and availability in large quantities (Kyriakopoulou et al. 2019). This high functionality comes at greater environmental and energy costs (Caraballo et al. 2024; van der Goot et al. 2016). Replacing these ingredients with less processed raw materials could reduce the environmental impact of food products (Singh et al. 2024).

Oilseed press cakes (PCs) are a by‐product of oil production. Currently they are used primarily for animal feed and energy production. If the demand were present, they would be available at an industrial scale for food production (Arrutia et al. 2020). With high levels of beneficial nutrients like fiber and various minor component phytochemicals (e.g., phenolics), plus high protein contents with a balanced amino acid profile, their valorization in meat alternatives would yield both nutritional and ecological benefits (Arntfield 2011; Arrutia et al. 2020).

The most widely used, well‐known technique to structure plant proteins and improve their textural properties is low‐moisture extrusion (Baune et al. 2021; Baune et al. 2022; Dekkers et al. 2018). In this process, a mix of protein‐rich flour and water is kneaded, sheared, and heated in a twin‐screw extruder, forming a cross‐linked protein network (Bouvier and Campanella 2014). Upon exiting the barrel through the die, the protein mass experiences a rapid pressure loss, leading to the formation of steam bubbles and an expansion of the extrudate (Lillford 2008). Subsequent cooling and evaporation lead to solidification, resulting in porous‐textured vegetable proteins (TVPs) that are used as meat analogues. Although quite common in food processing, extrusion processes are still not fully characterized and understood due to the high degree of interconnection between input and process parameters (Emin 2015, 2022). Empirical testing of the properties of PCs as potential new ingredients is therefore necessary.

The diverse composition of PCs can lead to several technological difficulties during processing (Asgar et al. 2010). Although a higher fat content in TVPs could be desirable to create juiciness, very few studies have covered the addition of oil in protein extrusion (Artz and Rao 1993; Gwiazda et al. 1987; Kendler et al. 2021; Kyriakopoulou et al. 2019). In fact, defatted flours are used in many cases (Antun et al. 2017; Cabrera et al. 1979; Caraballo et al. 2024), as oil is known to act as a lubricant in the extrusion process. Even at low concentrations (<2%), oil reduces the mechanical impact on the protein and impairs TVP structure formation, while fiber, also present at high levels in PCs, can modulate shear and expansion (Asgar et al. 2010; Camire 2001; Ilo et al. 2000; Opaluwa et al. 2024).

Several research groups have studied the inclusion of (usually low levels of) PCs in the formulation of extruded snacks in the context of enrichment or sustainability (Antun et al. 2017; Jaquez et al. 2014; Naseer et al. 2021; Philipp et al. 2017; Reyes‐Jáquez et al. 2012). Only recently have PCs attracted attention as ingredients for protein extrusion (Banjac et al. 2021; Baune et al. 2021; Choi et al. 2024; Martin et al. 2019; Caraballo et al. 2024; Singh et al. 2024; Vidal et al. 2022). To the authors’ knowledge, low‐moisture extrusion of pea protein PC mixtures has not yet been investigated.

This study characterizes how the different PCs affect the process and the resulting TVPs. Using PCs with different biological origins, compositions, and process histories, this study identifies shared techno‐functional properties of these ingredients and analyzes TVP properties with respect to potential usage in meat alternatives. The findings will enable manufacturers to assess the potential of different press cakes as ingredients in TVP formulations and to identify limiting raw material properties.

## 2 Materials and Methods

### 2.1 Materials

Press cakes (PCs) from six different plant sources (almond, coconut, flaxseed, pumpkin seed, rapeseed, and sunflower seed; see Table 1) were kindly provided by All Organic Treasures (Ltd., Wiggensbach/Allgäu, Germany), Henry Lamotte Oils (Ltd., Bremen, Germany), Kanow Mühle Spreewald (Golßen, Germany), Cargill (Inc., Minneapolis, MN, USA), and Ölmühle Moog (Ltd., Lommatzsch, Germany) and additionally purchased from OPW Ingredients (Ltd., Niederkrüchten, Germany) and Buxtrade (Ltd., Buxtehude, Germany). The PCs were delivered either as flour, pellets, or flakes and were milled through a 350 µm slit knife ring (Microcut MCH 20 K, Stephan Machinery Ltd.). Pea protein isolate Empro E 86 HV was kindly provided by Emsland‐Stärke (Ltd., Emlichheim, Germany). All chemicals used were of analytical grade. For reference, minced beef meat (REWE, Ltd., Cologne, Germany) and four different plant‐based mince products (based on soy, pea, and sunflower seeds; dmBio Sonnenblumenhack and dmBio Veggie Hack, dm‐drogerie markt, GmbH & Co. KG, Karlsruhe, Germany; Vantastic Granulat aus Soja, Vantastic, Ltd., Nabburg, Germany; Veganes Mühlen Hack, Rügenwalder Mühle Carl Müller, GmbH & Co. KG, Bad Zwischenahn, Germany) were purchased at local supermarkets. Three of these meat alternatives were retailed as dry TVPs to be soaked and drained prior to consumption, while one was sold as a pre‐hydrated, seasoned, and refrigerated convenience product.

**TABLE 1:** Composition of the press cakes.

Press cake	Producer	Protein conversion factor	Protein[%]	Fat[%]	Moisture[%]	Insoluble dietary fiber[%]	Soluble dietary fiber[%]	c_Max_[%]
Pumpkin seed, roasted	a	5.57	53.35 ± 0.04	10.84 ± 0.02	3.03 ± 0.05	11.05 ± 1.57	4.64 ± 0.05	100
Pumpkin seed	a	5.53	54.12 ± 0.03	10.09 ± 0.10	4.25 ± 0.07	11.31 ± 3.61	1.74 ± 1.06	100
Coconut	B	5.38	17.09 ± 0.03	16.92 ± 0.65	3.25 ± 0.10	31.26 ± 1.57	1.37 ± 0.25	50
Coconut	K	5.51	18.62 ± 0.07	6.73 ± 0.02	8.69 ± 0.08	40.58 ± 1.07	2.36 ± 0.54	51
Flaxseed	K	5.89	37.02 ± 0.54	20.61 ± 0.91	1.98 ± 0.07	21.23 ± 2.63	10.85 ± 3.00	69
Flaxseed	L	6.02	37.91 ± 0.18	5.84 ± 0.03	5.64 ± 0.03	23.05 ± 1.37	17.56 ± 4.18	69
Flaxseed	N	5.96	29.58 ± 0.44	13.14 ± 0.18	8.72 ± 0.03	26.03 ± 2.04	9.68 ± 2.35	59
Almond	a	5.67	43.91 ± 0.64	7.78 ± 0.25	6.52 ± 0.02	18.36 ± 1.40	2.48 ± 0.47	100
Almond	O	5.64	47.80 ± 0.22	8.81 ± 0.04	5.33 ± 0.01	18.17 ± 0.26	2.11 ± 0.54	94
Rapeseed	G	5.29	22.63 ± 0.26	17.83 ± 0.37	6.06 ± 0.01	29.94 ± 0.87	3.57 ± 0.48	55
Rapeseed	K	5.34	25.71 ± 0.17	12.13 ± 0.25	5.25 ± 0.24	28.22 ± 2.68	3.29 ± 0.40	61
Rapeseed	L	5.52	27.04 ± 0.84	10.21 ± 0.17	6.79 ± 0.22	32.46 ± 0.75	2.09 ± 0.43	59
Sunflower seed, shelled	a	5.60	41.87 ± 0.19	9.39 ± 0.12	7.00 ± 0.03	16.38 ± 1.99	2.04 ± 0.42	80
Sunflower seed, whole	a	5.26	22.52 ± 0.14	14.33 ± 0.23	7.42 ± 0.05	37.85 ± 0.48	4.25 ± 1.75	55
Sunflower seed, shelled	K	5.62	38.33 ± 0.13	20.99 ± 0.09	5.51 ± 0.13	13.82 ± 0.82	1.84 ± 0.69	69
Pea protein isolate	E	6.02	80.03 ± 0.20	0.12 ± 0.00	5.28 ± 0.00	1.51 ± 1.51	<1.0 ± 0.00	—

*Note*: Mean values of three measurements with standard deviations. c_max_ = maximum concentration of press cake in mixtures with pea protein isolate used in the extrusion experiments.

## 3 Methods

### 3.1 Raw Material Characterization

All raw materials were analyzed for their composition using standard methods. Fat content was determined gravimetrically by Soxhlet extraction in light petroleum (ISO 659:2009, equivalent to AOCS Am 2–93, DIN 2009). Moisture content was determined gravimetrically by drying in a drying chamber (Heraeus Holding, Ltd., Hanau, Germany) at 105°C until mass constancy (ISO 771:2021, equivalent to AOAC 926.12‐1926, ISO 2021). Nitrogen content was determined by combustion and subsequent nitrogen detection according to Dumas (Dumatherm, C. Gerhardt, GmbH & Co. KG, Königswinter, Germany; ISO 16634‐2, ISO 2016). Conversion factors to calculate protein content from nitrogen content were obtained by amino acid analysis via high‐performance liquid chromatography of selected raw materials, conducted by an external laboratory (SGS Germany, Ltd., Hamburg, Germany; ISO 13903:2005, DIN 2005; see data provided in Lallinger et al. 2025a). The content of insoluble and soluble dietary fiber was analyzed by simulated enzymatic digestion using an enzyme kit (K‐TDFR‐100A, Megazyme Ltd., Bray, Ireland), followed by filtration and precipitation in ethanol (AOAC 991.43, equivalent to AACC 32–07.01, Megazyme^2019^).

In addition to the composition, the particle sizes and water binding capacities of the PC flours were measured by static laser scatter analysis and gravimetric determination after centrifugation, respectively. As these properties did not have significant effects on extrusion, they are not discussed in this publication but can be viewed in the supplementary data set (see Lallinger et al. 2025a).

### 3.2 Low‐Moisture Extrusion

Press cake flours were mixed with pea protein isolate in a mixer (HCM 225, G&G Machinery Ltd., Qingdao, China) at three different levels based on total weights including moisture: 25% PC, 45% PC, and an individual highest concentration dependent on the PC's protein content, so that the mixture contained 50% protein (equaling 50 to 100% PC). The mixtures were extruded in a co‐rotating pilot‐scale twin‐screw extruder equipped with a gravimetric powder feeder (Werner & Pfleiderer ZSK 25 and K‐Tron, Coperion Ltd., Stuttgart, Germany) and a 2.5 mm hole die. The screws (diameter 25 mm, length 700 mm) were configured for high mechanical energy input, with compressing conveying elements in the front and three high‐shear zones (broad kneading elements followed by reverse conveying elements) in the last third of the screw length (separated by conveying elements). Based on pre‐trials, rotation velocity, powder mass flow, and pump water mass flow were kept constant at 480 min^−1^, 9.4 kg/h, and 1.18 kg/h (ca. 14% total moisture, depending on PC moisture), respectively. The first two barrel segments were cooled with cold tap water, while the remaining three were heated to 150°C in counterflow with an oil heating circuit (SINGLE Temperiertechnik GmbH, Hochdorf, Germany). Between sampling of different mixtures, the system was run for ca. 20 min of equilibration time. Screw speed (n) and torque (M) were measured by built‐in sensors; temperature (T_p_) and pressure at the die (p) were measured at the inner barrel wall 1 cm before the die plate with a thermocouple type J (Voltcraft, Wollerau, Switzerland) and a pressure transducer (PT411, Dynisco Instruments, LLC, Franklin, MA, USA), respectively. Measurements were recorded by the software (VEE 6.0 Pro, Agilent Technologies, Inc., Santa Clara, CA, USA) in 16 s intervals. The specific mechanical energy input (SME) was calculated according to Meuser, F., van Lengerich, B.H. (1984) from rotation speed, throughput (ṁ), and torque without idle torque (M‐M_empty_, both as a percentage of maximum torque at maximum power of the extruder P_max_, 8600 W; equation 1).

(1) $$\mathrm{SME}\;=\frac{\frac{480\;\mathrm{rpm}}{500\;\mathrm{rpm}}\cdot \left(M-M_{\mathrm{empty}}\right)\cdot 0.01\cdot P_{\max}}{\dot{m}}\;$$

The extruded TVP was granulated by a rotating knife and collected on metal mesh trays. The TVP was dried overnight at 40°C in a ventilated drying chamber (UT 6420, Heraeus Holding, Ltd., Hanau, Germany) and stored in plastic bags at ambient temperature until analysis.

### 3.3 Structural Properties of Extrudates

To elicit the macroscopic structure, the TVP sample was poured into a petri dish of ca. 10 cm diameter and placed in a white photo box illuminated by three white LED strips at the top to be photographed from above through an opening at the top. Brightness was adjusted so that the background would appear white, and the image was cropped (Photos, Microsoft Corporation, Inc., Redmond, WA, USA).

The microstructure of the TVP was examined by bright‐field microscopy. Texturized vegetable protein samples were hydrated in tap water at room temperature for approximately 20 min, frozen, and cut with a razor blade into slices of ca. 0.5–1 mm. The slices were thawed and photographed under a microscope (Eclipse E400 microscope fitted with DS‐Fi2 camera, Nikon Corporation, Inc., Tokyo, Japan) at 100x, 200x, and 400x magnification.

To measure the bulk density of the TVP, it was poured into a measuring cylinder cut at the 500 mL mark until exactly full without shaking. The cylinder was weighed after removal of excess above the opening (equation 2, Lallinger and Rauh 2024).

(2) $$\mathrm{density}\;=\frac{m_{\mathrm{TVP}}}{500\mathrm{ml}}\;$$

The sectional expansion index (SEI) was calculated according to Alvarez‐Martinez et al. (1988) from the TVP diameter measured with a caliper and the hole die diameter (d_TVP_ and d_die_, equation 3).

(3) $$\mathrm{SEI}\;={\left(\frac{d_{\mathrm{TVP}}}{d_{\mathrm{die}}}\right)}^{2}$$

Furthermore, the water absorption of the TVP was analyzed. Thirty grams of TVP were weighed into a tared 1 L beaker and left to soak for 15 min in 225 g of a boiling 1% sodium chloride solution made with tap water to mimic a meal preparation process. The TVP was strained on a metal mesh sieve for about 1 min. The water binding capacity (WBC) was calculated according to Samard et al. (2021) as brine uptake of the dry sample (equation 4).

(4) $$\mathrm{WBC}\;=\frac{m_{\mathrm{TVP},\mathrm{soaked}}-m_{\mathrm{TVP}}}{m_{\mathrm{TVP}}}$$

### 3.4 Preparation of Meat References

To create a meat reference sample, minced beef meat was pulled into small pieces of meat grinder die diameter (ca. 3 mm) and lengths of 5 to 10 mm. A separate portion of meat was formed into TVP‐sized balls (diameters 5 to 10 mm). Taking into account the high water content of the fresh samples compared to the TVP, 100 g of each sample geometry was soaked with 300 mL of boiling 1% NaCl solution for 15 min to denature the meat protein. The meat was drained on a sieve and cooled to room temperature before measuring the texture profile as described in the following section. A portion of the heated meat sample was freeze‐dried for measurement of the analogical breaking force to the TVP.

### 3.5 Texture Evaluation

The breaking force of the TVP was measured with a fracture test in a Kramer shear cell according to Philipp et al. (2017) with alterations described in a previous publication (Lallinger and Rauh 2024). Briefly, a pouring of TVP particles was crushed by ten parallel blunt blades, and the peak force of the resulting force‐displacement curve was defined as breaking force.

Textural properties of the hydrated PC‐based TVP and similarities with commercial meat alternatives and meat were analyzed by a double compression test (also known as texture profile analysis) modified for particle pourings (Barbut 2010; Ávila et al. 2014; Samard and Ryu 2019). The soaked TVP and commercial reference products prepared for WBC determination were cooled to room temperature. The sample was filled into a cylindrical sample container (5 cm diameter) so that the sample layer would be 15 mm ± 1.5 mm high. In a texture analysis device equipped with a 2 kN load cell (Z2.0, Zwick Roell, Inc., Ulm, Germany), the bulk sample was compressed to 75% of the initial height in two successive cycles by a cylindrical probe (diameter 2.5 cm) with 2 mm/s (0.05 N pre‐load; 5 s holding times at the decompression maximum). The counterforce was recorded by the texture analysis software (testXpert II, Zwick Roell), and the texture parameters were calculated from the force displacement curve according to Barbut (2010) with data analysis software (Origin Pro 2022b, OriginLab Corporation, Northampton, MA, USA).

Hardness is defined as the peak force of the measurement. Cohesiveness is described by the ratio of work conducted on the sample during the second and first compression cycles, including the decompression work. Springiness is defined as the ratio of the compression times in the first and second compression cycles. Gumminess and chewiness are calculated from the independent texture parameters (equations (5) and (6)).

(5) Gumminess=Hardness·Cohesiveness

(6) Chewiness=Gumminess·Springiness

The mass‐specific textural parameters were calculated by dividing the hardness, cohesiveness, etc. by the hydrated sample weights.

#### 3.5.1 Statistical Analyses

Raw material properties as well as pouring density, WBC, and breaking force of the TVP were analyzed in triplicate. For each extrusion experiment, T_p_, p, SI, M, and SME were measured as the mean of ten subsequent data sets (2.7 min). Three extrusion replications were conducted with each mixture. Texture profile analysis was conducted five times, and SEI was measured ten times for each sample.

As PC type and content were the only independent parameters of the experiment, while the natural properties of the raw materials were all dependent variables, scatter plots and correlation analyses according to Spearman were carried out to identify relations between PC and mixture composition and the resulting process output parameters and TVP properties. Correlation coefficients ρ and individual significance levels were calculated for each pair of parameters using a two‐sided significance test with a significance level of 0.05 (Origin Pro 2022b).

The influence of the factor PC content at the two predetermined levels, 25% and 45%, was analyzed by either the Mann‐Whitney test or the t‐test or Welch test, depending on the outcome of the Shapiro‐Wilk test and Levene test for each parameter. Non‐parametric tests were applied if the data or variances were not normally distributed (p‐value = 0.95).

## 4 Results and Discussion

### 4.1 Press Cake Composition

The composition of the PCs is presented in Table 1. The protein content of the investigated PCs ranges from 17.1 to 44.1% (Table 1), similar to literature values (Arntfield 2011). The protein/fiber ratio of the defatted seed is determined more by plant species than by cultivation: the ratio deviates by 65% between the different PC types, while the standard deviation within one PC type is on average only 9.2% (compare Wang et al. 2008). Pumpkin seed and almond flours have the highest protein content, indicating a high functionality in the extrusion process. The amount of dietary fiber lies between 20 and 45% and is strongly inversely correlated with the protein content (ρ_protein‐IDF_ = −0.91, p < 0.001). Due to the comparatively low protein content of coconut and rapeseed flours, higher proportions of added PPI are required to create a continuous protein matrix in the extruder (compare c_max_ in Table 1). Depending on the pressing equipment and technique used by each manufacturer, the oil contents vary between 5.8 and 21.0%, while the protein‐to‐fiber ratio is very similar in each PC category. For example, flaxseed PC extracted in a 5 t/d screw press (manufacturer N) and 20 kg batch hydraulic press (manufacturer K) contains 13.14% and 20.6% residual fat, respectively (relative difference 57%), while the protein‐to‐fiber ratio of flaxseed PC is 0.97 ± 0.17 (relative difference 17%).

As the PCs naturally contain more oil and fiber than the pea protein isolate, both of these contents increase with increasing PC percentage in the mixture. Depending on the PC type and concentration, the protein content per total mass of the TVP produced in this study ranges from 48 to 73%, which is very similar to the protein content of the commercial TVP products examined for comparison (49–71%; see Lallinger et al. 2025a). However, it should be noted that a nitrogen‐to‐protein conversion factor of 6.25 is common for food declaration purposes, which overestimates the protein content of plant products (compare Table 1).

### 4.2 Extrusion Process Response

**FIGURE 1:**
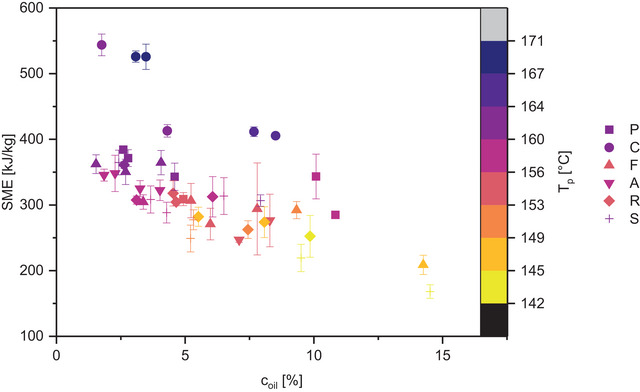
Specific mechanical energy input (SME) of textured vegetable protein with varied oil content (c_oil_). Symbol color represents temperature at the die (T_p_), and symbol shape corresponds with press cake type, marked with a capital first letter (pumpkin seed, coconut, flaxseed, almond, rapeseed, sunflower seed). Error bars represent standard deviations from three technical replicates.

During extrusion, the mechanical and thermal energy exerted on the dough by screw rotation and external heating is transferred into complex chemical reactions, viscosity, and temperature changes. Due to the functional interdependencies of these parameters, the extrusion responses T_p_, M, and SME correlate with each other (ρ_M‐Tp_ = 0.90, p‐value < 0.001, see Figure 1; Bouvier and Campanella 2014; Emin 2015). At similar extrusion conditions, higher values generally indicate more intense processing regimes.

The TVPs were extruded with SME 168 to 544 kJ/kg, T_p_ 142 to 171°C, and p 17 to 52 bar (Figure 1; for specific values, refer to Lallinger et al. 2025a). The higher the PC content of the mixture, the lower the affected process parameters M (ρ_M‐cPC_ = −0.48, p‐value < 0.001), SME (ρ_SME‐cPC_ = −0.45, p‐value = 0.002), and p (ρ_p‐cPC_ = −0.42, p‐value = 0.004) with significant differences between 25 and 45% PC (Mann‐Whitney test). On average, SME decreases from 335 ± 33 kJ/kg to 300 ± 35 kJ/kg when the PC content is increased from 25 to 45%. This is in agreement with a study on high‐moisture extrusion of sunflower‐soy blends in which SME was also found to decrease with increasing PC content (Singh et al. 2024). The type of PC also affects the processing. In particular, TVPs containing coconut stand out as compared to the other PCs. It is extruded at a significantly higher temperature (13°C above the average T_p_ of the other 45% PC‐TVP) and SME (40 and 47 kJ/kg, i.e., 43% and 56% above the average of other PCs at the same mass fraction; see full circle symbols in Figure 1). Moreover, temperature increases further with coconut PC content, while the opposite can be observed for all other PC types (without coconut: ρ_cPC‐Tp_ = −0.35, p‐value = 0.03).

The underlying cause of the effects of PC type and content seems to be the amount of oil in the formulation (ρ_SME‐cOil_ = −0.65, −0.76 if coconut is regarded as a outlier; ρ_Tp‐cOil_ = −0.59 (−0.69 without coconut), all p‐values < 0.001, Table 1). With each added percent of oil in the raw material mixture, T_p_ declines on average by 1.2°C and SME by 11.5 kJ/kg. Oil's lubricating and plasticizing (i.e. viscosity‐reducing) effects have been documented by several other authors (Bouvier and Campanella 2014; Ilo et al. 2000; Opaluwa et al. 2024; Singh et al. 2024) and also apply to low‐moisture TVP extrusion with PCs.

Although fiber can increase shear forces during extrusion, the insoluble fiber does not notably counteract the oil in the reduction of the process parameters (as observed for press cake‐starch blends by Martin et al. 2021) but instead contributes to viscosity and shear reduction through thermodynamic incompatibility of protein and polysaccharides and steric hindrance of protein cross‐linking (see Bouvier and Campanella 2014; Lillford 2008; Tolstoguzov 1993). However, this might be unique to coconut PCs. The extraordinarily large particles of the coconut PCs (see particle size distribution data in Lallinger et al. 2025a) and their low solubility likely significantly increase shear and subsequently temperature in the extruder.

In contrast to fiber, higher protein content has a slight mitigating effect on the SME reduction (ρ_SME‐cprotein_ = 0.31, p‐value = 0.04). In the process, the protein melt starts to cross‐link, thereby increasing the viscosity and thus the friction in the melt (Banjac et al. 2021; Bouvier and Campanella 2014). Apparently, this increase in viscosity influences friction to a greater extent than the presence of insoluble particles. However, due to the strong inverse correlation of protein content and fiber content in the PC, it is hard to differentiate between effects of low fiber and high protein content (compare to results from Singh et al. 2024).

As the mechanical energy input is diminished by PC addition (i.e., oil addition and protein reduction in the mixture), less energy is available for melting and cross‐linking of proteins, leading to the formation of protein networks with reduced stability. Extruded soy protein network stability increases with SME until ca. 610 kJ/kg (calculation based on melt viscosity; Yang et al. 2023), and all SME values in this study were below that threshold.

Analogous to torque and temperature, the addition of press cake decreases the pressure at the die (ρ_p‐cPC_ = 0.42, p‐value = 0.004), probably due to the reduction of viscosity with increasing oil and fiber contents (see supplementary data in repository: Lallinger et al. 2025a; compare Kendler et al. 2021; Singh et al. 2024).

A PC that contains high amounts of residual oil is probably less thoroughly pressed. This means that some oil can still be extracted when the forces on the cellular structure of the PC exceed its stability during extrusion (Vidal et al. 2022). As a result, fat extraction could be observed during extrusion processing of several PC mixtures, leading to a greasy‐looking TVP, semi‐continuous dripping of plant oil from the die, or formation of oil plugs.

### 4.3 Structural and Mechanical Properties of TVPs

The PC‐TVPs generated in this study ranged from a cereal‐like puffy structure to small flakes. Apart from separation of oil, separation of a dust fraction of intact PC particles or split‐off of smaller pieces from the main TVP was observed in some mixtures (images of all TVP types are available in the corresponding repository data: Lallinger et al. 2025b). Textured vegetable protein of 45% or more PC resembles the pea, sunflower, and pumpkin seed TVPs presented by Baune et al. (2021).

**FIGURE 2:**
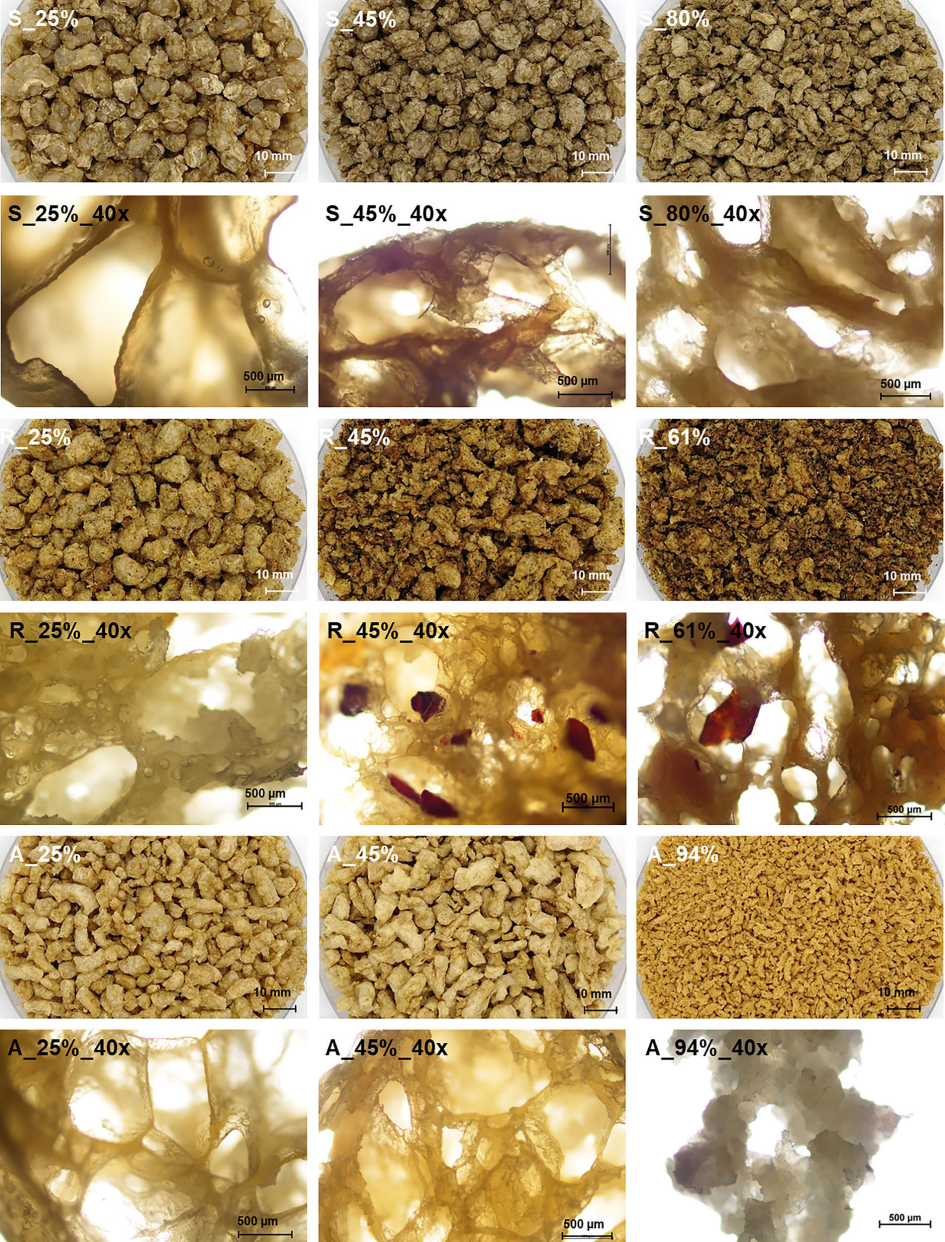
Macroscopic and microscopic (40x = 40x objective with 10x ocular) images of TVP from different mixtures of pea protein and press cakes (S = shelled sunflower seed from producer A, R = rapeseed from producer K, and A = almond from producer O). Left column: 25% press cake; center column: 45% press cake; right column: 80%, 61%, or 94% press cake.

**FIGURE 3:**
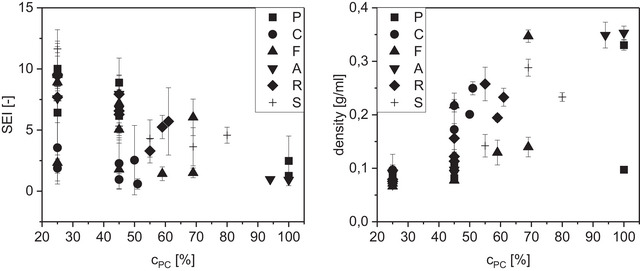
Sectional expansion index (SEI, left) and density (right) of TVP plotted against the press cake content (mixed with pea protein isolate). Press cake types represented by symbol shape (P—pumpkin seed, C—coconut, F—flaxseed, A—almond, R—rapeseed, S—sunflower seed). Error bars represent standard deviations of three technical replicates.

Figure 2 juxtaposes the macroscopic and microscopic structures of the selected PC‐TVPs from different mixtures. While there is a clear influence of the individual PCs on the appearance of the TVPs, the granulated particles become increasingly small, rough, and open‐pored with higher PC content. At 25% PC, the foam cell walls are very thin and separate large polyhedron‐like air pockets of more than 2 mm diameter in sunflower and almond TVP, while rapeseed TVP—containing less protein—is more compact (S_25%_40x, A_25%_40x, and R_25%_40x in Figure 2; compare expansion and density in Figure 3). At 45% PC, the air bubbles are more irregular in shape and size, and the lamellae in between are thicker. In rapeseed TVP (R_45%_40x, Figure 2), particles of the dark seed hulls are visible. Similar seed hull fragments are discernible in TVP from flaxseed and sunflower seed with husks (see Lallinger et al. 2025b). At 50% total protein, the TVP is rough and irregularly shaped, with even thicker foam cell walls, and the disconnected fragments indicate that some protein films were torn during expansion. Coconut, roasted pumpkin seed, and almond TVPs with the highest PC content are not properly textured under the extrusion conditions used here. Instead, they take on a crumb‐ or grit‐like structure (e.g., Figure 2, A_94% and A_94%_40x) with sizes below the die diameter (SEI<1, see Figure 3). However, unpublished experiments show that pure roasted pumpkin seed PC can also be properly texturized by adjustment of extrusion parameters (e.g., reduction of throughput to 6.5 to 7 kg/h at 140 to 145°C barrel temperature).

The influence of the PCs on the TVP structure stems from their modification of both shear stress and melt temperature, as well as the introduction of insoluble particles to the formulation. In the observed range, higher SMEs imply a higher energy input available for protein interaction formation, which can stabilize the protein film that is expanded at the die (Lillford 2008; Yang et al. 2023). Higher temperatures reduce viscosity and increase steam pressure, favoring higher expansion ratios but also a higher tendency to rupture. Thus, both process parameters define the porosity and density of the TVP.

As the oil introduced by the PCs reduces SME and temperature (see Figure 1), the protein network is weakened and the die pressure is lower. As a result, the bubbles formed are smaller and collapse more easily than in PC‐free formulations. The insoluble fiber particles act as nuclei for bubbles and stress hot spots in the protein films, where they rip during expansion (Bouvier and Campanella 2014; Lillford 2008), leading to finer bubble size distribution and more open pores, especially in pumpkin and sunflower seed TVPs. Consequently, the SEI drops from an average of 7.6 at 25% PC content to 3.0 at 50% total protein (significant difference between 25 and 45%; Figure 3).

The emergence of a powder side stream or even the total disintegration of the TVP would only be expected above 50 or even 60% of insoluble particles in the continuous phase not contributing to melt network formation (Bouvier and Campanella 2014). Therefore, this phenomenon can only be explained by the combined influences of oil, fiber, and protein effects. Apart from the torque reduction, oil can coat particles (fiber or aggregated protein) and inhibit their adherence to the protein film, further weakening the melt structure (Lillford 2008). Altogether, thermodynamic incompatibility, steric hindrance, and coating, lubrication, and lack of continuous matrix lead to the formation of denser TVP with increasing PC content (between 0.07 g/ml and 0.35 g/ml; ρ_cPC‐density_ = 0.84, p < 0.0001, Figure 3).

**FIGURE 4:**
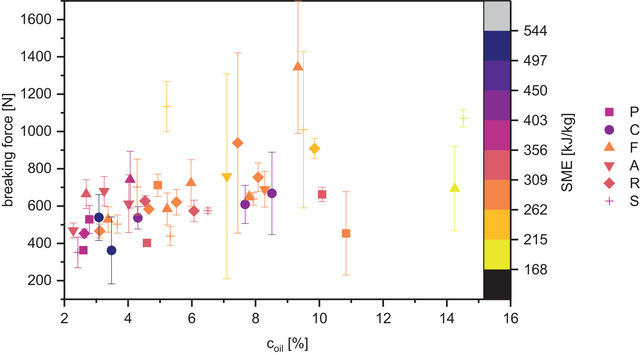
Breaking force of textured vegetable protein (TVP) in Kramer shear test. Symbol color represents specific mechanical energy input during TVP extrusion (SME); symbol shape corresponds with press cake type, marked with a capital first letter (pumpkin seed, coconut, flaxseed, almond, rapeseed, and sunflower seed). Error bars represent standard deviations of three technical replicates.

The altered TVP structure directly impacts its hardness: on average, the peak force in the fracture test is higher if the PC content and consequently oil content is higher (ρ_F‐cPC_ = 0.58, p‐value < 0.0001, see Figure 4), i.e., when the TVP is denser (ρ_F‐density_ = 0.59, p‐value < 0.0001) and contains thicker lamellae (Figure 2). Similar transitions from crispy to crunchy texture have been reported for fiber‐enriched extrudates by Bouvier and Campanella (2014), Caraballo et al. (2024), and Lillford (2008). However, if the density of the TVP is only due to the grit‐like structure and absence of bubbles in poorly textured samples (coconut, roasted pumpkin seed, almond), the TVP cannot withstand high forces despite the density. The low protein network stability of unexpanded extrudates might also explain the low hardness of rapeseed PC extrudates observed by Martin et al. (2021). If the mechanical energy exerted on the melt is insufficient for continuous protein network formation, the mechanical stability of the TVP is low (Singh et al. 2024). Consequently, the breaking force does not continue to increase with PC content above 10% of oil in the raw material composition or below ca. 250 kJ/kg SME (Figure 4).

The particle diameters of the commercial reference products are between 3.5 and 4 mm. With the hole die used in this study, similar sizes are obtained in >45% PC. TVP high in rapeseed or sunflower as well as TVP with 45% flaxseed shows the highest structural resemblance to commercial TVP in size and breaking force (850 to 1210 N), while the TVPs containing lower amounts of PCs are so expanded and fragile that they more closely resemble cereal or snack products than minced meat analogues.

### 4.4 Texture of Hydrated TVP

Dry TVP is usually hydrated before consumption to mimic the juiciness and water content of meat. As the water content is a main factor determining the viscoelastic behavior of food hydrocolloids, the water uptake of the dry TVP was measured (WBC). On average, the 25% PC‐TVP takes up 2.75 times its weight in water (Table 2), which is close to the absorption of the reference products (2.68 ± 0.03 to 2.97 ± 0.05 g/g). Textured vegetable proteins with higher oil content and lower protein content—which consequentially have a higher density—show a lower WBC (mean WBC of 45% PC‐TVP 2.41 g/g, ρ_cOil‐WBC_ = −0.48, p‐value < 0.001; ρ_cprotein‐WBC_ = 0.43, p‐value = 0.004; ρ_density‐WBC_ = −0.50, p‐value < 0.001). Collinear with these properties, PC and IDF correlate with reduced WBC. These results indicate that most of the water absorbed into the TVP is bound as capillary water in pores, such as in TVP from soy and gluten (Samard et al. 2021). Therefore, denser TVP with a smaller volume fraction of pores can bind less water despite the higher fiber content, which increases the swelling capacity of the raw material mixture (Table 2, see supplementary material Lallinger et al. 2025a). Furthermore, diffusion of water into the lamellae and closed pores takes longer through thicker lamellae, and excess oil on the surface might form a hydrophobic barrier. This could leave some parts dry or incompletely hydrated after the soaking time of 15 min.

**TABLE 2:** Water binding capacities (WBC) and texture parameters of TVP from press cakes (PCs) mixed with pea protein at different concentrations (c_PC_).

Formulation				Absolute texture profile parameters				Mass specific texture profile parameters			
PC		c_PC_	WBC	Hardness	Cohesiveness	Springiness	Chewiness	Hardness	Cohesiveness	Springiness	Chewiness
		[%]	[g/g]	[N]	[‐]	[‐]	[N]	[N/g]	[10^−2^/g]	[10^−2^/g]	[N/g]
A	(a)	25	2.78 ± 0.27	25 ± 8	0.64 ± 0.03	0.84 ± 0.03	13.6 ± 4.8	2.6 ± 0.8	6.7 ± 0.3	8.8 ± 0.3	1.4 ± 0.5
A	(a)	45	2.79 ± 0.07	39 ± 11	0.61 ± 0.03	0.82 ± 0.00	19.5 ± 5.1	4.0 ± 1.2	6.3 ± 0.5	8.4 ± 0.3	2.0 ± 0.5
A	(a)	100	4.92 ± 0.76	2 ± 0	0.54 ± 0.06	0.97 ± 0.09	1.2 ± 0.1	0.1 ± 0.0	2.0 ± 0.3	3.5 ± 0.2	0.0 ± 0.0
A	(O)	25	2.94 ± 0.30	42 ± 8	0.69 ± 0.05	0.88 ± 0.02	26.0 ± 6.9	3.8 ± 0.7	6.2 ± 0.5	7.9 ± 0.2	2.3 ± 0.6
A	(O)	45	2.50 ± 0.29	48 ± 9	0.66 ± 0.04	0.85 ± 0.05	27.4 ± 8.3	4.6 ± 1.2	6.3 ± 0.8	8.1 ± 0.9	2.6 ± 0.9
A	(O)	94	3.41 ± 0.39	24 ± 12	0.36 ± 0.06	1.02 ± 0.13	9.1 ± 5.9	1.0 ± 0.5	1.5 ± 0.3	4.3 ± 0.4	0.4 ± 0.3
C	(B)	25	2.70 ± 0.20	39 ± 2	0.72 ± 0.01	0.87 ± 0.02	24.5 ± 1.2	4.4 ± 0.8	8.1 ± 1.0	9.7 ± 1.3	2.7 ± 0.4
C	(B)	45	2.07 ± 0.10	56 ± 5	0.66 ± 0.01	0.83 ± 0.04	30.7 ± 3.0	5.2 ± 0.4	6.1 ± 0.5	7.7 ± 0.3	2.8 ± 0.1
C	(B)	50	2.09 ± 0.09	57 ± 7	0.64 ± 0.03	0.81 ± 0.03	29.5 ± 6.0	4.7 ± 0.9	5.3 ± 0.8	6.7 ± 0.7	2.5 ± 0.6
C	(K)	25	2.51 ± 0.12	45 ± 2	0.75 ± 0.03	0.89 ± 0.08	29.7 ± 2.9	4.5 ± 0.2	7.4 ± 0.7	8.8 ± 1.1	3.0 ± 0.4
C	(K)	45	2.10 ± 0.08	73 ± 6	0.65 ± 0.01	0.79 ± 0.02	37.2 ± 2.2	6.0 ± 0.9	5.3 ± 0.3	6.4 ± 0.2	3.1 ± 0.4
C	(K)	51	2.26 ± 0.09	73 ± 3	0.62 ± 0.02	0.75 ± 0.03	34.0 ± 1.3	5.7 ± 0.4	4.8 ± 0.2	5.9 ± 0.2	2.6 ± 0.1
F	(K)	25	2.38 ± 0.40	33 ± 5	0.61 ± 0.02	0.88 ± 0.08	17.8 ± 1.5	3.5 ± 0.4	6.5 ± 0.8	9.5 ± 2.3	1.9 ± 0.2
F	(K)	45	1.75 ± 0.06	26 ± 11	0.41 ± 0.03	0.79 ± 0.01	8.5 ± 2.9	2.5 ± 1.1	3.8 ± 0.2	7.3 ± 0.2	0.8 ± 0.3
F	(K)	69	3.04 ± 0.07	5 ± 1	0.36 ± 0.14	0.91 ± 0.05	1.5 ± 0.2	0.3 ± 0.1	1.8 ± 0.7	4.5 ± 0.3	0.1 ± 0.0
F	(L)	25	2.96 ± 0.19	36 ± 3	0.59 ± 0.05	0.86 ± 0.02	18.5 ± 3.1	3.4 ± 0.3	5.5 ± 0.7	8.0 ± 0.5	1.7 ± 0.3
F	(L)	45	2.92 ± 0.11	36 ± 3	0.59 ± 0.05	0.86 ± 0.02	18.5 ± 3.1	3.4 ± 0.3	5.5 ± 0.7	8.0 ± 0.5	1.7 ± 0.3
F	(L)	69	2.62 ± 0.05	34 ± 6	0.53 ± 0.06	0.81 ± 0.02	14.8 ± 4.5	2.8 ± 0.5	4.4 ± 0.4	6.6 ± 0.2	1.2 ± 0.4
F	(M)	25	2.61 ± 0.05	20 ± 1	0.57 ± 0.04	0.84 ± 0.04	9.5 ± 0.5	2.0 ± 0.1	5.8 ± 0.4	8.5 ± 0.4	1.0 ± 0.0
F	(M)	45	2.46 ± 0.09	15 ± 0	0.42 ± 0.04	0.76 ± 0.08	5.0 ± 1.0	1.4 ± 0.0	3.9 ± 0.4	7.0 ± 0.7	0.5 ± 0.1
F	(M)	59	2.34 ± 0.15	17 ± 2	0.48 ± 0.04	0.81 ± 0.03	6.4 ± 0.8	1.0 ± 0.1	2.8 ± 0.2	4.7 ± 0.2	0.4 ± 0.0
P	(a)	25	2.76 ± 0.13	44 ± 6	0.72 ± 0.01	0.87 ± 0.02	27.2 ± 3.6	4.3 ± 0.6	7.1 ± 0.1	8.6 ± 0.1	2.7 ± 0.4
P	(a)	45	2.10 ± 0.05	62 ± 6	0.71 ± 0.02	0.87 ± 0.03	38.5 ± 5.1	7.0 ± 0.7	8.0 ± 0.1	9.7 ± 0.1	4.3 ± 0.5
P	(a)	100	1.34 ± 0.10	75 ± 22	0.56 ± 0.03	0.72 ± 0.04	29.5 ± 9.1	6.6 ± 2.9	5.1 ± 1.3	6.5 ± 1.6	2.6 ± 1.2
P	(a)	25	3.13 ± 0.21	29 ± 1	0.68 ± 0.01	0.83 ± 0.07	16.2 ± 1.4	3.5 ± 0.4	8.3 ± 0.7	10.1 ± 1.3	2.0 ± 0.3
P	(a)	45	3.22 ± 0.16	39 ± 3	0.72 ± 0.01	0.87 ± 0.02	24.5 ± 2.2	4.6 ± 0.6	8.4 ± 0.4	10.2 ± 0.5	2.9 ± 0.4
P	(a)	100	2.33 ± 0.02	72 ± 7	0.73 ± 0.04	0.85 ± 0.03	44.1 ± 1.9	7.5 ± 0.2	7.7 ± 1.1	9.0 ± 1.2	4.6 ± 0.3
R	(G)	25	2.54 ± 0.06	33 ± 14	0.69 ± 0.06	0.88 ± 0.03	20.3 ± 10.5	3.4 ± 1.5	7.1 ± 0.6	9.1 ± 0.3	2.1 ± 1.1
R	(G)	45	2.20 ± 0.05	31 ± 12	0.63 ± 0.02	0.84 ± 0.01	16.6 ± 5.9	3.3 ± 1.3	6.8 ± 0.2	9.0 ± 0.1	1.8 ± 0.6
R	(G)	55	1.87 ± 0.13	34 ± 11	0.60 ± 0.04	0.80 ± 0.01	16.7 ± 6.5	3.6 ± 1.2	6.3 ± 0.5	8.4 ± 0.1	1.8 ± 0.7
R	(K)	25	2.66 ± 0.20	44 ± 4	0.72 ± 0.01	0.88 ± 0.02	27.6 ± 2.7	4.4 ± 0.4	7.2 ± 0.6	8.8 ± 0.7	2.8 ± 0.3
R	(K)	45	2.39 ± 0.23	55 ± 13	0.73 ± 0.03	0.88 ± 0.02	35.0 ± 6.7	5.2 ± 1.0	7.0 ± 0.9	8.3 ± 0.8	3.3 ± 0.5
R	(K)	61	1.97 ± 0.06	56 ± 8	0.68 ± 0.08	0.86 ± 0.04	32.7 ± 3.7	4.9 ± 0.8	5.4 ± 0.6	6.9 ± 0.7	2.6 ± 0.7
R	(L)	25	2.61 ± 0.32	42 ± 6	0.68 ± 0.07	0.83 ± 0.04	23.9 ± 5.5	4.5 ± 0.7	7.4 ± 2.0	8.9 ± 2.3	2.8 ± 0.4
R	(L)	45	2.50 ± 0.04	44 ± 2	0.68 ± 0.08	0.86 ± 0.03	25.9 ± 4.8	3.8 ± 0.0	6.0 ± 0.3	7.4 ± 0.4	2.2 ± 0.4
R	(L)	59	2.04 ± 0.07	46 ± 2	0.62 ± 0.05	0.83 ± 0.02	23.9 ± 3.3	4.0 ± 0.3	5.5 ± 0.6	7.3 ± 0.5	2.1 ± 0.4
S	(a)	25	3.59 ± 0.09	31 ± 10	0.70 ± 0.07	0.88 ± 0.02	19.5 ± 8.1	2.8 ± 0.3	6.4 ± 1.1	8.2 ± 1.8	1.7 ± 0.4
S	(a)	45	2.87 ± 0.21	46 ± 7	0.68 ± 0.04	0.86 ± 0.04	26.8 ± 6.2	4.2 ± 0.3	6.2 ± 0.5	7.9 ± 0.5	2.4 ± 0.4
S	(a)	80	2.20 ± 0.04	48 ± 4	0.64 ± 0.03	0.82 ± 0.03	25.0 ± 3.0	3.8 ± 0.4	5.0 ± 0.6	6.5 ± 0.7	2.0 ± 0.1
S	(K)	25	2.97 ± 0.12	31 ± 7	0.71 ± 0.06	0.88 ± 0.02	19.5 ± 5.9	2.8 ± 0.5	6.5 ± 0.7	8.1 ± 0.6	1.8 ± 0.5
S	(K)	45	1.90 ± 0.22	54 ± 7	0.64 ± 0.04	0.88 ± 0.01	30.9 ± 4.0	5.3 ± 0.5	6.3 ± 0.5	8.6 ± 1.0	3.0 ± 0.2
S	(K)	69	1.72 ± 0.11	61 ± 8	0.57 ± 0.02	0.81 ± 0.02	28.7 ± 4.0	4.8 ± 0.7	4.4 ± 0.3	6.3 ± 0.4	2.2 ± 0.3
S	(a)	25	3.25 ± 0.35	47 ± 6	0.69 ± 0.07	0.85 ± 0.05	27.6 ± 6.4	4.2 ± 0.6	6.2 ± 0.6	7.7 ± 0.3	2.5 ± 0.6
S	(a)	45	2.37 ± 0.15	48 ± 3	0.70 ± 0.06	0.82 ± 0.02	27.4 ± 4.5	4.0 ± 2.1	6.0 ± 3.2	6.9 ± 3.5	2.4 ± 1.4
S	(a)	55	2.22 ± 0.08	63 ± 2	0.68 ± 0.06	0.83 ± 0.01	35.4 ± 4.0	5.7 ± 0.6	6.1 ± 1.0	7.4 ± 0.7	3.2 ± 0.6

*Note*: Press cake type is represented by the capital first letter (almond, coconut, flaxseed, pumpkin seed, rapeseed, sunflower seed), and the producer is in brackets (one‐letter code). Mean values and standard deviations of three technical replicates.

The results of the instrumental texture profile analysis provide insights into the potential sensory perception of the TVPs. Because the TVPs have a wide range of densities and particle sizes, they were measured as loose pouring to enable a representative, reproducible, and comparable sampling technique. However, the distribution of gaps between the particles is hard to control, leading to high deviations of the textural parameters, obscuring correlations, and impeding their interpretation.

When the TVP is chewed, the water stored in the pores is easily released, creating a juicy impression (Caraballo et al. 2024). Thus, it barely contributes to the counterforce measured in a double compression test. This makes TVP with higher WBC less hard (ρ_WBC‐hardness_ = −0.54, p‐value < 0.001). However, no uniform trend across all PC types could be observed concerning the hardness (i.e., the absolute maximum bite force (Table 2)). Textured vegetable proteins made with pumpkin seed, coconut, and sunflower seed PCs are harder at higher PC levels and thus fiber content, but the opposite can be observed in TVP from flaxseed PC (in agreement with results published earlier; Lallinger and Rauh 2024). Possibly, the high content of soluble fiber in flaxseed leads to the formation of a different microstructure in these TVP. While the PC content has been reported to increase apparent firmness of soaked TVP from some PC legume protein blends, it decreases firmness in HMMA (Caraballo et al. 2024; Singh et al. 2024).

**FIGURE 5:**
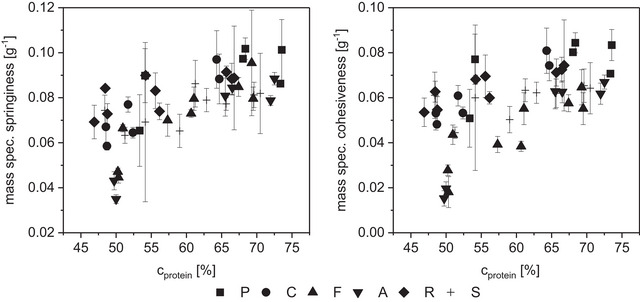
Mass‐specific springiness (left) and cohesiveness (right) over protein concentration in the TVP raw material mixture. Symbol shape corresponds with press cake type, marked with the capital first letter (pumpkin seed, coconut, flaxseed, almond, rapeseed, and sunflower seed). Error bars represent standard deviations of three technical replicates.

Cohesiveness and springiness of the TVP represent structural integrity and elasticity of the sample and range between 0.36 and 0.75 and 0.72 and 1.02, respectively (Table 2). Both characteristics are influenced by compositional parameters. Textured vegetable proteins with a higher PC content are less springy and require less work to disintegrate (ρ_cPC‐springiness_ = −0.33, p‐value = 0.03; ρ_cPC‐massspec.springiness_ = −0.66, p‐value < 0.001, significant difference between 25% and 45% PC; ρ_cPC‐cohesiveness_ = −0.48, ρ_cPC‐massspec.cohesiveness_ = −0.62, p‐values < 0.001). The springiness and cohesiveness of the TVP increase with the protein content of the mixture, even more so when normalized for sample density (ρ_cprotein‐massspec.cohesiveness_ = 0.62, ρ_cprotein‐massspec.springiness_ = 0.69, p‐values < 0.001; Figure 5). In coherence with the microscopic analysis, TVPs with higher protein content form stronger protein networks with more bonds between protein molecules and fewer imperfections. In a rehydrated state, this gel‐like network has elastic properties. Therefore, springiness and cohesiveness are high when PC content is low. In contrast, continuous protein phases that are interrupted by fiber particles and oil droplets or films are more prone to break at these phase boundaries during compression, similar to the effect seen during expansion (compare Lillford 2008).

**FIGURE 6:**
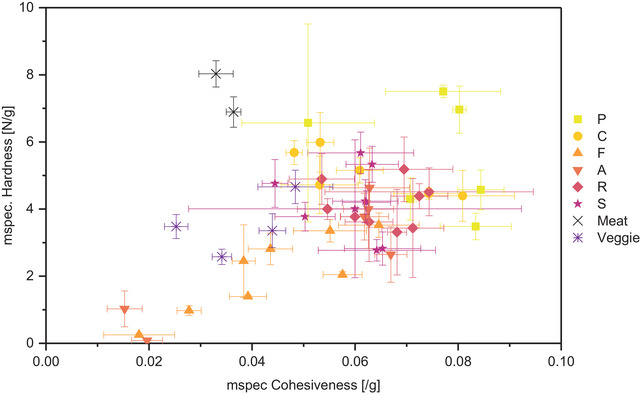
Textural properties mass‐specific hardness and cohesiveness (normalized by sample weight) of hydrated textured vegetable protein from press cake (PC) and pea protein. Symbol color and shape correspond with press cake (marked with a capital first letter: pumpkin seed, coconut, flaxseed, almond, rapeseed, and sunflower seed) or reference product type. Error bars represent standard deviations of three technical (PC‐TVP) or five measurement replicates (reference products), respectively.

As demonstrated in Figure 6, the texture of cooked minced meat is characterized by a high initial hardness (6.9 to 8.0 ± 0.45 N/g), combined with a low mass specific and total springiness and cohesiveness (springiness 0.73–0.8 ± 0.04 or 0.056 ± 0.003 g^−1^; cohesiveness 0.47 ± 0.04 or 0.03 ± 0.003 g^−1^). In other words, the firm meat structure is irreversibly destroyed during the first bite, so that the second bite requires much less work (47% of the first bite). While these hardness and springiness values closely match with those reported for sausage and cooked turkey, a cohesiveness of around 0.85 was measured for these meat types (Paredes et al. 2022). This disparity could be attributable to the differences in the measuring method. The cohesiveness for the commercial meat alternative products is similar to or a little higher than that of minced meat (0.45 ± 0.04 to 0.67 ± 0.04), while the force required for the first bite is only half of meat's value (2.6 ± 0.23 to 4.6 ± 0.50 N/g).

Most of the PC‐TVPs have a hardness that ranges between the analyzed meat and commercial plant‐based mince products, but they have much higher cohesiveness and springiness (cohesiveness average 0.68 for 25% PC, Figure 6). Normalized for the different densities, the mass‐specific cohesiveness at 25% PC is twice as high as that of meat (0.068 g^−1^). The high springiness of TVP with low PC content leads to a spongy, rubbery texture, while meat is more firm and chewy. Thus, TVP with a higher PC content is more suited to mimicking cooked minced meat, as the structure is less elastic. Of all PC‐TVPs, the crumbly, poorly textured TVP with high roasted pumpkin seed content, as well as one coconut and one sunflower extrudate, have values that most closely resemble that of meat. With their high powder fractions, however, they seem unsuitable as products. Although the hardness is much less pronounced, some flaxseed (45‐69% PC) and sunflower seed (80% PC) samples have similar cohesiveness values and therefore match the commercial veggie products (Table 2). Unroasted 100% pumpkin seed PC‐TVP, on the other hand, has a high hardness (like meat), but its cohesiveness has to be reduced to increase the similarity to meat. Combining all parameters of the texture profile analysis, chewiness of high‐PC TVPs with sunflower seed, coconut, rapeseed, and pumpkin seed shows the most meat‐like values (30–45 N, Table 2; Lallinger et al. 2025a).

## 5 Conclusion

The results of this study explain how the composition of PCs affects the properties of TVPs made with them. The protein‐to‐fiber ratio of each PC type is stable across suppliers, while the remaining oil content depends on the pressing technique. The resulting composition of the raw material is highly relevant for the extrusion properties. Therefore, simple specification information allows for reasonable estimation of how suitable a PC raw material might be for TVP production.

The presence of oil combined with a lack of protein reduces torque, SME, and T_p_, in the extrusion of PC pea protein isolate mixtures. With increasing PC content, the TVP becomes denser, with smaller, more evenly sized, and open pores due to the lubricating and coating effects of oil combined with the steric and thermodynamic effects of fiber from the natural PC composition. Remaining above a total protein concentration of 50% in the raw material mixture, TVPs comprising up to 100% pumpkin seed PC, 45% coconut PC, 69% flaxseed PC, 45% almond PC, 61% rapeseed PC, or 80% sunflower seed PC were successfully extruded.

With increasing addition of PC in the formulation, the extrudate structure changes from a puffed‐up, cereal‐like product to unevenly shaped chunks and scraps. The expanded, crispy TVP with low PC content (25%) could be used in high‐protein cereal or snack bars, while TVP with high PC content (45–100%) can serve as a minced meat alternative because it is chewier and less spongy when soaked. Therefore, TVP with elevated PC content shows a higher textural resemblance to meat.

Further optimization of the extrusion parameters would be required to achieve the desired textural properties for each PC, but the current study proves that PCs can be used as protein ingredients for TVPs.

## Nomenclature

a: all organic treasures (producer)
A: almond
B: buxtrade (producer)
c: concentration
C: coconut
d: diameter
E: Emsland Stärke (producer)
F: flaxseed
G: Cargill (producer)
K: Kanowmühle Sagritz (producer)
L: Henry Lamotte Oils (producer)
ṁ: throughput
m_TVP_
: mass of TVP sample
M: torque
N: Ölmühle Moog (producer)
n: rotation velocity
O: OPW Ingredients (producer)
p: pressure
P: pumpkin seed
P_max_
: maximum power output of extruder
PC: press cake
R: rapeseed
ρ: Spearman correlation coefficient
S: sunflower seed
SEI: sectional expansion index
SME: specific mechanical energy input
T_p_
: product temperature at the die
TVP: textured vegetable protein

## Author Contributions

**Luise Lallinger**: conceptualization, data curation, investigation, project administration, visualization, writing – original draft, writing – review and editing, formal analysis. **Torres Gomez Alejandra Maria**: formal analysis, investigation, writing – original draft. **Alejandra Maria**: formal analysis, investigation, writing – original draft. **Jonas Niksch**: writing – original draft, investigation, formal analysis. **Cornelia Rauh**: conceptualization, writing – review and editing, funding acquisition, supervision, resources.

## Conflicts of Interest

The authors declare no conflicts of interest.

## Data Availability

All data discussed in this article, as well as supplementary data (e.g., color values of TVP), are publicly available in a repository (Lallinger et al. 2025a, 2025b).

## References

[jfds70471-bib-0001] Alcorta, A. , A. Porta , A. Tárrega , M. D. Alvarez , and M. P. Vaquero . 2021. “Foods for Plant‐Based Diets: Challenges and Innovations.” Foods 10, no. 2: 292–309. 10.3390/foods10020293. 33535684 PMC7912826

[jfds70471-bib-0002] Alvarez‐Martinez, L. , K. P. Kondury , and J. M. Harper . 1988. “A General Model for Expansion of Extruded Products.” Journal of Food Science 53, no. 2: 609–615. 10.1111/j.1365-2621.1988.tb07768.x.

[jfds70471-bib-0003] Antun, J. , A. Đurđica , J. Stela , et al. 2017. “Optimisation of Extrusion Variables for the Production of Corn Snack Products Enriched With Defatted Hemp Cake.” Czech Journal of Food Sciences 35, no. 6: 507–516. 10.17221/83/2017-CJFS.

[jfds70471-bib-0004] Arntfield, S. D. 2011. “Canola and Other Oilseed Proteins.” In Handbook of Food Proteins, edited by G. Phillips and P. A. Williams , 289–315. Woodhead Publishing. 10.1533/9780857093639.289.

[jfds70471-bib-0005] Arrutia, F. , E. Binner , P. Williams , and K. W. Waldron . 2020. “Oilseeds Beyond Oil: Press Cakes and Meals Supplying Global Protein Requirements.” Trends in Food Science & Technology 100: 88–102. 10.1016/j.tifs.2020.03.044.

[jfds70471-bib-0006] Artz, W. E. , and S. K. Rao . 1993. “Lipid Oxidation in Extruded Products.” In ACS Symposium Series. Thermally Generated Flavors, edited by T. H. Parliment , M. J. Morello , and R. J. McGorrin , Vol. 543. 296–314. American Chemical Society. 10.1021/bk-1994-0543.ch024.

[jfds70471-bib-0007] Asgar, M. A. , A. Fazilah , N. Huda , R. Bhat , and A. A. Karim . 2010. “Nonmeat Protein Alternatives as Meat Extenders and Meat Analogs.” Comprehensive Reviews in Food Science and Food Safety 9, no. 5: 513–529. 10.1111/j.1541-4337.2010.00124.x. 33467834

[jfds70471-bib-0008] Banjac, V. , Đ. Vukmirović , L. Pezo , V. Draganovic , O. Đuragić , and R. Čolović . 2021. “Impact of Variability in Protein Content of Sunflower Meal on the Extrusion Process and Physical Quality of the Extruded Salmonid Feed.” Journal of Food Process Engineering 44, no. 3: e13640. 10.1111/jfpe.13640.

[jfds70471-bib-0009] Barbut, S. 2010. “Chapter 8 Texture Analysis.” In Sensory Analysis of Foods of Animal Origin, edited by L. M. Nollet and F. Toldra , 121–131. CRC Press.

[jfds70471-bib-0010] Baune, M. ‑ C. , A. ‑ L. Jeske , A. Profeta , et al. 2021. “Effect of Plant Protein Extrudates on Hybrid Meatballs—Changes in Nutritional Composition and Sustainability.” Future Foods 4: 1–11. 10.1016/j.fufo.2021.100081.

[jfds70471-bib-0011] Baune, M. ‑ C. , N. Terjung , M. Ç. Tülbek , and F. Boukid . 2022. “Textured Vegetable Proteins (TVP): Future Foods Standing on Their Merits as Meat Alternatives.” Future Foods 6: 100181. 10.1016/j.fufo.2022.100181.

[jfds70471-bib-0012] Bouvier, J. ‑ M. , and O. H Campanella . 2014. Extrusion Processing Technology: Food and Non‐Food Biomaterials. Wiley‐Blackwell.

[jfds70471-bib-0013] Cabrera, J. , L. E. Zapata , T. S. Buckle , I. Ben‐Gera , A. M. Sandoval , and I. Shomer . 1979. “Production of Textured Vegetable Protein From Cottonseed Flours.” Journal of Food Science 44, no. 3: 826–829. 10.1111/j.1365-2621.1979.tb08512.x.

[jfds70471-bib-0014] Camire, M. E. 2001. “Extrusion and Nutritional Quality.” In Extrusion Cooking, 108–129. Elsevier. 10.1533/9781855736313.1.108.

[jfds70471-bib-0015] Choi, H. W. , J. Hahn , H. ‑ S. Kim , and Y. J Choi . 2024. “Thermorheological Properties and Structural Characteristics of Soy and Pumpkin Seed Protein Blends for High‐Moisture Meat Analogs.” Food Chemistry 464, no. Pt 3: 1–9. 10.1016/j.foodchem.2024.141768. 39520886

[jfds70471-bib-0016] Clark, M. A. , M. Springmann , J. Hill , and D. Tilman . 2019. “Multiple Health and Environmental Impacts of Foods.” Proceedings of the National Academy of Sciences of the United States of America 116, no. 46: 23357–23362. 10.1073/pnas.1906908116. 31659030 PMC6859310

[jfds70471-bib-0017] Dekkers, B. L. , R. M. Boom , and A. J. van der Goot . 2018. “Structuring Processes for Meat Analogues.” Trends in Food Science & Technology 81: 25–36. 10.1016/j.tifs.2018.08.011.

[jfds70471-bib-0018] DIN (Deutsches Institut für Normung) . 2005. Futtermittel—Bestimmung des Aminosäuregehalts *(ISO 13903:2005): Deutsche Fassung EN ISO 13903:2005 (DIN EN ISO 13903)*. DIN.

[jfds70471-bib-0019] DIN (Deutsches Institut für Normung) . 2009. Ölsamen—Bestimmung des Ölgehaltes (Referenzverfahren) (DIN EN ISO 659). Beuth.

[jfds70471-bib-0020] Emin, M. A. 2015. “Modeling Extrusion Processes.” In Modeling Food Processing Operations, 235–253. Elsevier. 10.1016/B978-1-78242-284-6.00009-X.

[jfds70471-bib-0021] Emin, M. A. 2022. “Key Technological Advances of Extrusion Processing.” In Food Engineering Innovations Across the Food Supply Chain, 131–148. Elsevier. 10.1016/B978-0-12-821292-9.00005-4.

[jfds70471-bib-0022] Gwiazda, S. , A. Noguchi , &, and K. Saio . 1987. “Microstructural Studies of Texturized Vegetable Protein Products: Effects of Oil Addition and Transformation of Raw Materials in Various Sections of a Twin Screw Extruder.” Food Structure 6, no. 1: 8.

[jfds70471-bib-0023] Ilo, S. , R. Schoenlechner , and E. Berghofe . 2000. “Role of Lipids in the Extrusion Cooking Processes.” Grasas Y Aceites 51, no. 1‐2: 97–110. 10.3989/gya.2000.v51.i1-2.410.

[jfds70471-bib-0024] International Organization for Standardization (ISO) . 2021. Oilseed Meals — Determination of Moisture and Volatile Matter Content (ISO 771:2021). ISO.

[jfds70471-bib-0025] International Organization for Standardization (ISO) . 2016. *Food products — Determination of the Total Nitrogen Content by Combustion According to the Dumas Principle and Calculation of the Crude Protein Content*: *Part 2: Cereals, Pulses and Milled Cereal Products (ISO 16634‐2)* . ISO.

[jfds70471-bib-0026] Jaquez, R. D. , F. Casillas , N. Flores , et al. 2014. “Effect of Glandless Cottonseed Meal Content on the Microstructure of Extruded Corn‐Based Snacks.” Advances in Food Science 36: 125–130.

[jfds70471-bib-0027] Kendler, C. , A. Duchardt , H. P. Karbstein , and M. A. Emin . 2021. “Effect of Oil Content and Oil Addition Point on the Extrusion Processing of Wheat Gluten‐Based Meat Analogues.” Foods (Basel, Switzerland) 10, no. 4: 697. 10.3390/foods10040697. 33805896 PMC8064384

[jfds70471-bib-0028] Kyriakopoulou, K. , B. Dekkers , and A. J. van der Goot . 2019. “Plant‐Based Meat Analogues.” In Sustainable Meat Production and Processing, edited by C. M. Galanakis , 103–126. Academic Press. 10.1016/B978-0-12-814874-7.00006-7.

[jfds70471-bib-0029] Lallinger, L. , and C. Rauh . 2024. “Textured Vegetable Protein From Flaxseed Press Cake and Pea—syntonizing Chemical and Structural Properties.” Future Foods 10: 100489. 10.1016/j.fufo.2024.100489.

[jfds70471-bib-0030] Lallinger, L. , A. M. Torres Gomez , and J. Niksch . 2025a. Oil Seed Press Cakes in Textured Vegetable Protein—Raw Material, Processing and TVP Properties. Technische Universität Berlin. 10.14279/depositonce-22303.

[jfds70471-bib-0031] Lallinger, L. , A. M. Torres Gomez , and J. Niksch . 2025b. Textured Vegetable Protein from Press Cakes—Macroscopic and Microscopic Images . Dataset. DepositOnce. https://depositonce.tu‐berlin.de/handle/11303/24324.

[jfds70471-bib-0032] Aguilera, J. M. , P. J. Lillford , eds. 2008. “Extrusion.” In Food Material Science. Principles and Practice, 415–435. Springer. https://link.springer.com/book/10.1007%2F978‐0‐387‐71947‐4.

[jfds70471-bib-0033] Martin, A. , R. Osen , A. Greiling , H. P. Karbstein , and M. A. Emin . 2019. “Effect of Rapeseed Press Cake and Peel on the Extruder Response and Physical Pellet Quality in Extruded Fish Feed.” Aquaculture 512: 1–11. 10.1016/j.aquaculture.2019.734316.

[jfds70471-bib-0034] Martin, A. , R. Osen , H. P. Karbstein , and M. A. Emin . 2021. “Linking Expansion Behaviour of Extruded Potato Starch/Rapeseed Press Cake Blends to Rheological and Technofunctional Properties.” Polymers 13, no. 2: 215. 10.3390/polym13020215. 33435355 PMC7826698

[jfds70471-bib-0035] Megazyme . 2019. Total Dietary Fiber Assay Kit . Bray Business Park. https://secure.megazyme.com/Total‐Dietary‐Fiber‐Assay‐Kit.

[jfds70471-bib-0036] Meuser, F. , B. H. , van Lengerich . 1984. “Systems Analytical Model for the Extrusion of Starches.” In Thermal Processing and Quality of Foods, edited by P., Zeuthen , et al., 175–179. Elsevier Applied Science Publishers.

[jfds70471-bib-0037] Morejón Caraballo, S. , S. V. Fischer , K. Masztalerz , et al. 2024. “Low Moisture Texturised Protein From Sunflower Press Cake.” International Journal of Food Science & Technology 59, no. 11: 8236–8247. 10.1111/ijfs.17513.

[jfds70471-bib-0038] Naseer, B. , V. Sharma , S. Z. Hussain , and J. Bora . 2021. “Development of Functional Snack Food From Almond Press Cake and Pearl Millet Flour.” Letters in Applied NanoBioScience 11, no. 1: 3191–3207. 10.33263/LIANBS111.31913207.

[jfds70471-bib-0039] Opaluwa, C. , S. Deskovski , H. P. Karbstein , and M. A. Emin . 2024. “Effect of Oil on the Rheological Properties and Reaction Behavior of Highly Concentrated Wheat Gluten Under Conditions Relevant to High Moisture Extrusion.” Future Foods 9: 1–12. 10.1016/j.fufo.2024.100307.

[jfds70471-bib-0040] Paredes, J. , D. Cortizo‐Lacalle , A. M. Imaz , J. Aldazabal , and M. Vila . 2022. “Application of Texture Analysis Methods for the Characterization of Cultured Meat.” Scientific Reports 12, no. 1: 3898. 10.1038/s41598-022-07785-1. 35273231 PMC8913703

[jfds70471-bib-0041] Philipp, C. , R. Buckow , P. Silcock , and I. Oey . 2017. “Instrumental and Sensory Properties of Pea Protein‐Fortified Extruded Rice Snacks.” Food Research International (Ottawa, Ont.) 102: 658–665. 10.1016/j.foodres.2017.09.048. 29195997

[jfds70471-bib-0042] Poore, J. , and T. Nemecek . 2018. “Reducing Food's Environmental Impacts Through Producers and Consumers.” Science (New York) 360, no. 6392: 987–992. 10.1126/science.aaq0216. 29853680

[jfds70471-bib-0043] Reyes‐Jáquez, D. , F. Casillas , N. Flores , et al. 2012. “The Effect of Glandless Cottonseed Meal Content and Process Parameters on the Functional Properties of Snacks During Extrusion Cooking.” Food and Nutrition Sciences 3, no. 12: 1716–1725. 10.4236/fns.2012.312225.

[jfds70471-bib-0044] Romerode Ávila, M. D. , M. Isabel Cambero , J. A. Ordóñez et al. 2014. “Rheological Behaviour of Commercial Cooked Meat Products Evaluated by Tensile Test and Texture Profile Analysis (TPA).” Meat Science 98, no. 2: 310–315. 10.1016/j.meatsci.2014.05.003. 24880977

[jfds70471-bib-0045] Samard, S. , T. ‑ T. Maung , B. ‑ Y. Gu , M. ‑ H. Kim , and G. ‑ H Ryu . 2021. “Influences of Extrusion Parameters on Physicochemical Properties of Textured Vegetable Proteins and Its Meatless Burger Patty.” Food Science and Biotechnology 30, no. 3: 395–403. 10.1007/s10068-021-00879-y. 33868750 PMC8017041

[jfds70471-bib-0046] Samard, S. , and G. ‑ H Ryu . 2019. “A Comparison of Physicochemical Characteristics, Texture, and Structure of Meat Analogue and Meats.” Journal of the Science of Food and Agriculture 99, no. 6: 2708–2715. 10.1002/jsfa.9438. 30350409

[jfds70471-bib-0047] Schösler, H. , J. d. Boer , and J. J Boersema . 2012. “Can We Cut out the Meat of the Dish?' Constructing Consumer‐Oriented Pathways Towards Meat Substitution.” Appetite 58, no. 1: 39–47. 10.1016/j.appet.2011.09.009. 21983048

[jfds70471-bib-0048] Singh, R. , A. G. A. Sá , S. Sharma , et al. 2024. “Effects of Feed Moisture Content on the Physical and Nutritional Quality Attributes of Sunflower Meal‐Based High‐Moisture Meat Analogues.” Food and Bioprocess Technology 17, no. 7: 1897–1913. 10.1007/s11947-023-03225-8. 38939448 PMC11199254

[jfds70471-bib-0049] Singh, R. , S. Langyan , S. Sangwan , et al. 2022. “Protein for Human Consumption from Oilseed Cakes: A Review.” Frontiers in Sustainable Food Systems 6: 856401. 10.3389/fsufs.2022.856401.

[jfds70471-bib-0050] Tolstoguzov, V. B. 1993. “Thermoplastic Extrusion—the Mechanism of the Formation of Extrudate Structure and Properties.” Journal of the American Oil Chemists' Society 70, no. 4: 417–424.

[jfds70471-bib-0051] van der Goot, A. J. , P. J. M. Pelgrom , J. A. M. Berghout , et al. 2016. “Concepts for Further Sustainable Production of Foods.” Journal of Food Engineering 168: 42–51. 10.1016/j.jfoodeng.2015.07.010.

[jfds70471-bib-0052] Vidal, N. P. , L. Roman , V. S. Swaraj , et al. 2022. “Enhancing the Nutritional Value of Cold‐Pressed Oilseed Cakes Through Extrusion Cooking.” Innovative Food Science & Emerging Technologies 77: 1–11. 10.1016/j.ifset.2022.102956.

[jfds70471-bib-0053] Wang, Z. ‑ H. , S. ‑ X. Li , and S. Malhi . 2008. “Effects of Fertilization and Other Agronomic Measures on Nutritional Quality of Crops.” Journal of the Science of Food and Agriculture 88, no. 1: 7–23. 10.1002/jsfa.3084.

[jfds70471-bib-0054] Yang, L. , Z. Ying , H. Li , et al. 2023. “Extrusion Production of Textured Soybean Protein: the Effect of Energy Input on Structure and Volatile Beany Flavor Substances.” Food Chemistry 405, no. Pt A: 1–11. 10.1016/j.foodchem.2022.134728. 36370569

